# An optimized procedure greatly improves EST vector contamination removal

**DOI:** 10.1186/1471-2164-8-416

**Published:** 2007-11-13

**Authors:** Yi-An Chen, Chang-Chun Lin, Chin-Di Wang, Huan-Bin Wu, Pei-Ing Hwang

**Affiliations:** 1 Bioinformatics Core Laboratory, Agricultural Biotechnology Research Center, Academia Sinica, Taipei, Taiwan; 2 Lab of Mathematics in Biology, Institute of Statistical Sciences, Academia Sinica, Taipei, Taiwan

## Abstract

**Background:**

The enormous amount of sequence data available in the public domain database has been a gold mine for researchers exploring various themes in life sciences, and hence the quality of such data is of serious concern to researchers. Removal of vector contamination is one of the most significant operations to obtain accurate sequence data containing only a cDNA insert from the basecalls output by an automatic DNA sequencer. Popular bioinformatics programs to accomplish vector trimming include LUCY, cross_match and SeqClean.

**Results:**

In a recent study, where the program SeqClean was used to remove vector contamination from our test set of EST data compiled through various library construction systems, however, a significant number of errors remained after preliminary trimming. These errors were later almost completely corrected by simply using a re-linearized form of the cloning vector to compare against the target ESTs. The modified trimming procedure for SeqClean was also compared with the trimming efficiency of the other two popular programs, LUCY2, and cross_match. Using SeqClean with a re-linearized form of the cloning vector significantly surpassed the other two programs in all tested conditions, while the performance of the other two programs was not influenced by the modified procedure. Vector contamination in dbEST was also investigated in this study: 2203 out of the 48212 ESTs sampled from dbEST (2007-04-18 freeze) were found to match sequences in UNIVEC.

**Conclusion:**

Vector contamination remains a serious concern to the data quality in the public sequence database nowadays. Based on the results presented here, we feel that our modified procedure with SeqClean should be recommended to all researchers for the task of vector removal from EST or genomic sequences.

## Background

The enormous amount of sequence data available in the public domain database has been a gold mine for researchers exploring various themes in life sciences. However, the quality of such data greatly affects subsequent analyses and hence is of serious concern to researchers. Data quality problems with the international sequence databanks have been reported previously [1,2], for example, multiple representations of the same gene, vector contamination in a non-vector sequence [3,4] and erroneous annotation of the exon/intron site in a gene [5].

Expressed Sequence Tags (ESTs) [6] are single reads of partially sequenced cDNA fragments. EST sequences have been applied to studies on gene prediction, alternative splicing, differential gene expression in specific tissues, etc.. More than half of the files in GenBank are EST data (based on release 154) [7], which were often produced in a batch sequencing project by automated processes. A computational processing procedure is therefore needed to obtain a reliable sequence which solely represents the molecular information of the cDNA. There is already a well-established procedure for EST sequence processing: trace data clean-up followed by removal of vector contamination. Various efficient bioinformatics tools such as phred [8,9] have been made available on the internet for the purpose of trace data clean-up. Additionally, offline software like cross_match [10] by Phil Green, SeqClean [11] and LUCY [12] from TIGR [13], or the online program VecScreen [14] available at NCBI [15] are popular computer programs for vector contamination detection and/or removal.

Cross_match was implemented based on a restricted Smith-Waterman algorithm [16]. The program executes a highly sensitive and efficient alignment to identify similarity between the two sequences under comparison, and it requires additional computer processing to remove the identified vector-like region from the target sequences. Cross_match has been incorporated into some EST processing packages such as PartiGene [17], a pipeline which integrates several bioinformatics programs to accomplish processes including sequence clean-up, EST clustering, assembly and functional annotation.

LUCY [12] searches for the greatest number of high quality subsequences in the query sequence according to the quality value of each base, which has been computed by a chromatogram base-calling program, such as phred [8,9], on the trace data. LUCY then identifies the vector spliced or ligated to the input sequence based on a depth-first search algorithm, which gives a stringency of 95% minimal sequence similarity. The program finally removes the identified vector sequence from the input.

SeqClean [11] utilizes BLAST [18] to remove any sequence highly similar (minimum 94% identity by default) to a given list of vectors, adaptors, primers, or linker sequences that is located within 30% of total EST from the 3' or 5' end of the sequences to be trimmed. SeqClean also removes polyA repeats and applies low complexity filtering (in addition to performing sequence alignment) to identify similar vector segments in the target EST. Following the trimming process, the program "trashes" those sequences which are shorter than 100 nucleotides or contain more than 3% undetermined bases (N) in the resulting sequences.

Using BLAST, VecScreen can identify vector and primer contamination by comparing the input EST sequences at preset parameters against the Univec [19] database, which contains most of the commonly used commercial vectors and primers. However, several limitations are associated with this procedure. For example, a batch search function is not provided at the VecScreen website and furthermore, it does not remove those segments from the screened ESTs.

The SeqClean program was initially selected for use in our study. This program can be downloaded from the TIGR website for free, and it has been used in several EST projects [20-23] as well as a genome sequencing project [24]. A large number of the EST sequences deposited in the public domain database may have been subjected to vector contamination removal by SeqClean. Here, we report an anomaly in the SeqClean trimming process and provide a refined procedure that greatly improves the trimming efficiency of SeqClean. Trimming efficiency was also compared to the other programs mentioned above. In addition, the error rate in the EST division of GenBank [3,4] was evaluated.

## Results

### Causes of errors in EST vector removal

**Figure 1:**
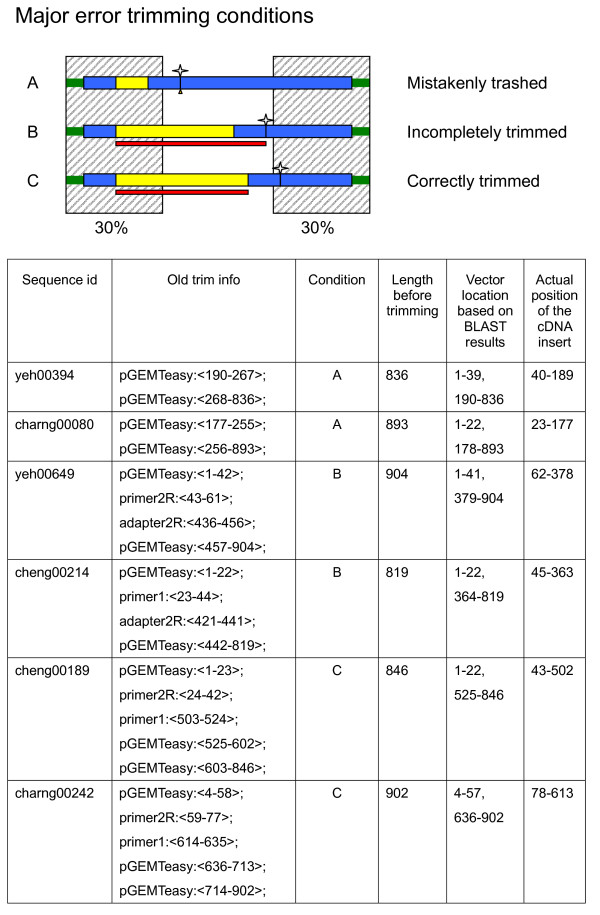
**Illustration of some trimming details**. The shaded area highlights the range covering the 30% from either end of the EST. According to the original SeqClean design, the vector contaminant is recognized only if some or all of the similar vector sequence is identified within this range. The boxes in blue indicate the vector-derived sequence. The yellow open boxes represent cDNA inserts and the green bars show the low quality regions. The small stars indicate where the number 1 base is located by CVS coordinates. The boxes in red specify the product of SeqClean trimming. Comments for each of the three listed trimming situations are denoted to their right. Condition A indicates those ESTs which were mistakenly trashed. Condition B shows incomplete trimming and condition C is an example of correct trimming. Example ESTs corresponding to each of the three conditions are shown in the table below, where the position numbering followed the coordinates of the untrimmed EST sequences.

We performed vector trimming (using the SeqClean program downloaded from TIGR) to clean up thousands of EST sequences derived by automatic sequencing of randomly picked clones of tomato cDNA libraries prepared at our facility (manuscript in preparation). After initial SeqClean trimming, some sequences, when examined with BLAST against the nucleotide sequence of their corresponding cloning vectors at scores above 50, were still found to contain a significant amount of unwanted vector sequences within the trimmed segments. The problem was investigated further by closely examining some of the imperfectly trimmed ESTs. Representative examples are shown in Figure 1. With condition A in Figure 1, SeqClean overlooked two consecutive DNA segments in an EST including a vector fragment and a cDNA insert located within 30% range of the 5' end. The entire EST sequence was therefore considered to contain no insert and was trashed by the program, even though the cDNA insert was longer than 100 bases. For other EST sequences, such as in condition B, the entire vector fragment, extending from the base numbered 1 in its conventional expression to the ligation site, was mistakenly recognized as part of the cDNA insert by the SeqClean program and remained untrimmed by SeqClean processing.

Based on the observations above, a hypothesis was made that the trimming program neglected the circular form of the vector DNA which resulted in erroneous trimming of some of the EST sequences.

Most cloning vectors exist in a circular form in nature. However, to conveniently manage these molecules electronically, the sequences are usually displayed in linear form by opening the circular DNA at a specific position where the base is either numbered 1 (if located downstream) or the final base (if it is located upstream). Discontinuous numbering of the circular DNA sequence across the junction may lead to misinterpretation in bioinformatic analyses if the analysis program does not take this virtual transformation into consideration.

### Marked improvement made to vector trimming with modified procedure

**Figure 2:**
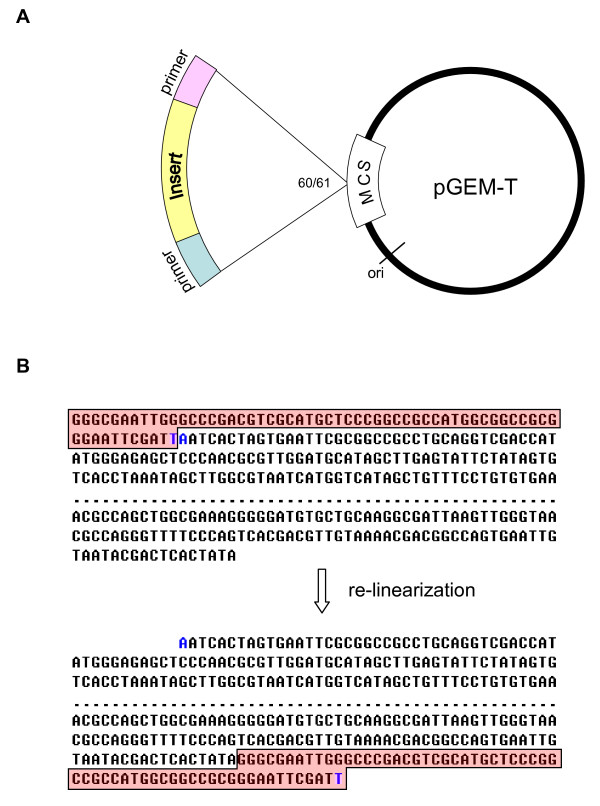
**Re-linearization of vector pGEM-T at its cloning site**. A. Simplified map of vector pGEM-T. The insert DNA of interest was cloned into position between bases 60 and 61. The primers were introduced with the DNA insert during cDNA preparation carried out in a wet lab. B. Vector sequence of pGEM-T before and after re-linearization Bases 1–198 and 2899–3015 were expressed. The omitted nucleotides are expressed as dotted lines. Additional nucleotides TA (colored in blue) were appended to the vector at position 60 during a wet-lab experimental procedure. The letters in pink boxes (bases 1 ~ 60 plus the appended T) were moved electronically to the end of the sequence for vector re-linearization.

A test of the above hypothesis was made with the SeqClean trimming process by using a modified vector sequence which had been re-linearized by opening the circular vector DNA at its ligation site, as depicted in Figure 2. The resulting DNA sequences, denoted as re-linearized vector sequences (RVSs) in the following parts of this paper, with the conventional sequence representation of a vector denoted as CVSs. Following SeqClean processing against either form of the vector sequences, the trimmed ESTs were subjected to BLAST alignment against the sequence of their corresponding cloning vector to examine the completeness of the automated vector trimming process.

Using 6,035 ESTs produced in the research laboratories in our institute as a test set for vector removal by SeqClean, vector contamination rate was reduced from 18.5% to 0.0% by trimming against RVS. Furthermore, 96 of the ESTs were "rescued" by this modified procedure (Table 1) after closely examining the trimming details provided in the output.

**Table 1:** Effects of SeqClean on phred-treated EST against CVS or RVS form of the vector sequence

	SeqClean	
	
	CVS	RVS
Number of ESTs to trim	6035	6035
Number trashed	239	143
Number of incompletely trimmed ESTs	1118	2
% incomplete trimming^a^	18.5%	0.0%

^a ^% of incomplete trimming was obtained by dividing the number of incompletely trimmed ESTs by the number of ESTs to trim. For example, the percentage of incompletely trimmed ESTs in the CVS column was calculated as: 1118/6035 × 100%.

### Comparison of SeqClean, LUCY2 and Cross_match on CVS or RVS vectors

To further investigate whether our modified procedure is generally applicable to most cloning vectors with SeqClean processing, and how this procedure could affect the performance of other software, two other popular trimming programs, cross_match and LUCY2, and an additional three sets of ESTs downloaded from Trace Archive [25] at NCBI were included in this study (Table 2).

**Table 2:** Cloning vectors, primers and adaptors used for ESTs in this study

Vector (cutting site)	Primer/adaptor	No. seq	Source of EST
PGEM-Teasy (60–61)	primer1, primer2R, adapter1, adapter2R	2882	ABRC laboratories
pTriplEx2 (571–581)	ToTom	2318	ABRC laboratories
pCR21TOPO (294–295)	primer1, primer2R, adapter1, adapter2R	5	ABRC laboratories
pZL1 (239–253)	*Not*I primer-adapter, *Sal*I adapter	700	ABRC laboratories
pT7Blue (95–96)^a^	*Not*I primer-adapter, *Sal*I adapter	130	ABRC laboratories
pCMV-SPORT6.1 (774–1222)		3000	trace archive at NCBI
pT7T3-PAC (200–240)	*Eco*RI adaptors	3000	trace archive at NCBI
pDNR-LIB (77–298)	Sfi-(dT)_30_; AAGCAGTGGTATCAACG CAGAGTGGCC	3000	trace archive at NCBI

^a ^Only 14 of the 130 ESTs cloned into pT7Blue in this test used the indicated adapter while the remaining 116 used no adapters.

The chromatographic data of all test ESTs was cleaned with the program phred prior to processing by one of the trimming programs (see methods). The trimming performance of the three programs against CVS or RVS vectors were all compared in terms of number of incompletely-trimmed ESTs, number of low quality (e.g. shorter than 100 bases) ESTs discarded by a trimming program, and the average lengths of trimmed ESTs. As LUCY was designed to find the longest high quality subsequences in the query sequence according to the quality value of each base, it can take a quality file in addition to a sequence file of the target EST as input. Therefore, LUCY was tested taking either form of the input (the column LUCY2 versus LUCY2(qul) in Table 3) in this study.

**Table 3:** Effect of vector forms in different trimming programs

		**Trimming Programs**			
		**SeqClean**	**cross_match**	**LUCY2**	**LUCY2(qul)**
CVS	# resulting ESTs^a^	14729	14870	14817	14808
	# removed ESTs	306	165	218	227
	# incompletely trimmed ESTs	1128	586	155	156
	% contamination	7.50%	3.90%	1.03%	1.04%
	average length of trimmed ESTs	551.4 ± 163.4	560.7 ± 175.4	546.3 ± 176.7	539.7 ± 175.6

RVS	# resulting ESTs^a^	14825	14870	14793	14785
	# removed ESTs	210	165	242	250
	# incompletely trimmed ESTs	12	637	190	193
	% contamination	0.08%	4.14%	1.26%	1.28%
	average length of trimmed ESTs	543.0 ± 172.1	563.5 ± 171.8	546.9 ± 176.7	540.1 ± 175.6

^a ^number of ESTs whose length is equal to or greater than 100 bases after being processed by a vector trimming program as indicated

Among all the tested conditions (three programs and two vector forms), SeqClean trimming against RVS markedly outperformed all the other conditions, delivering the lowest error rate without removing any additional ESTs (Table 3 and Additional file 1 for details).

It appears that directly trimming the phred-cleaned sequences with LUCY2 gives a slightly better result than using quality files as input on all parameters tested (Table 3). Without modifying the vector form, LUCY2 in combination with phred cleaning appeared to surpass the other two programs, giving the lowest error rate without reducing the amount of useful information (Table 3). When trimming against RVSs, LUCY2 and cross_match did not seem to benefit from this modification as much as SeqClean did. It is thus proposed that the design flaw found with SeqClean very likely did not exist in the other two programs, hence rearrangement of the vector sequence may have little influence on these programs' performance.

To gain a closer look at the error distribution over different vectors, the results of incomplete trimming caused by each of the three programs were grouped by the vector used (Table 4). The errors caused by SeqClean mainly occurred to ESTs cloned into the vector pGEM-Teasy, which accounted for eight cDNA libraries constructed with the similar experimental procedure [26]. Though the ESTs cloned in pTriplEX2 were cleaned very well by SeqClean under either form of the vector, pTriplEX2 ESTs were incompletely trimmed to a significant extent by both cross_match and LUCY2 (Table 4). The effect of the two vector forms on cross-match trimming seemed to be stochastically distributed among the ESTs cloned with different vectors (Table 4). Cross_match, based on a highly sensitive alignment algorithm, however, does not provide a cleaned segment of the target sequence after identifying segments of vector/adaptor/primer similar regions (The exact number of incompletely trimmed ESTs under each vector set is shown in the Additional file 2).

**Table 4:** Effect of vectors on vector trimming performance by three programs

Vector	#EST tested	Trimming Programs							
		**SeqClean**		**Cross_match**		**LUCY2**		**LUCY2**(qul)	
		
		CVS	RVS	CVS	RVS	CVS	RVS	CVS	RVS
PCMV. SPORT-6.1	3000	0.03%	0.03%	3.17%	3.17%	0.30%	0.27%	0.17%	0.17%
PT7T3PAC	3000	0.00%	0.00%	0.23%	0.77%	0.10%	0.07%	0.03%	0.03%
PDNR-LIB	3000	0.30%	0.30%	1.17%	1.17%	0.00%	0.00%	0.03%	0.03%
pGEM-Teasy	2882	38.38%	0.03%	6.45%	7.15%	0.52%	0.52%	0.31%	0.31%
pTriplEx2	2318	0.04%	0.04%	9.66%	9.66%	4.66%	4.87%	5.31%	5.52%
pCR21TOPO	5	0.00%	0.00%	0.00%	0.00%	0.00%	0.00%	0.00%	0.00%
pZL1	700	1.43%	0.00%	3.86%	3.86%	2.71%	2.71%	2.29%	2.29%
pT7Blue	130	0.77%	0.00%	9.23%	9.23%	0.77%	25.38%	0.77%	25.38%

Total	15035	7.50%	0.08%	3.90%	4.14%	1.03%	1.26%	1.04%	1.28%

Contamination rates of ESTs resulted from vector trimming under indicated conditions were grouped by the cloning vectors. The ratio was expressed in percentage showing the proportion of the ESTs using a specific cloning vector remained incompletely cleaned.

The program leaves users to decide on which vector-free segments in the target sequence processed by cross-match should be output as the final product. For users who wish to conduct vector trimming with cross_match, software like PartiGene is recommended for it provides cleaned-up versions of the processed sequences. In this comparison of the trimming efficiency with the other two programs, we simply took the longest segment as the trimmed product.

### Investigation of vector contamination rate of dbEST

As we observed different error rates caused by the vector trimming programs tested above, we were curious to see how much overall contamination remained in the dbEST [27]. A total of 48212 entries was randomly sampled from the entire dbEST (2007-04-18 freeze) by retrieving every 600^th ^EST entry from the database. These ESTs, were then subjected to BLAST analysis against UniVec database, which contains a redundancy-reduced set of representative sequences of commonly used cloning vectors. The same filtering criteria used above were applied to the BLAST results with E-value cutoff adjusted for the size of UniVec. 2203 (4.6%) of the sampled ESTs were observed to match some vector or primer/adaptor/linker sequence in Univec.

Though the analysis above provides a rough estimation of the extent of vector contamination in dbEST, it could be an overestimation of the error rate. To assess the vector contamination more accurately, the sampled ESTs needed to be compared against the sequence of their specific cloning vectors. As some of the EST entries in dbEST lacked vector information, some of the vector data were incorrect and some of the vectors' sequences were difficult to obtain, a subset of 35363 sampled ESTs which had been cloned into the 22 most prevalent vectors, derived from 4728 libraries and submitted by around 865 research groups in dbEST (see Methods for more detail), were used for this part of our study. Following BLAST analysis, 575 of the 35363 dbEST-derived ESTs showed a significant match to the sequence of their cloning vectors, giving a vector contamination rate of 1.63% with an average match length of 51.0 bases. The 575 ESTs which had significant vector remaining were cloned from 231 cDNA libraries, using 22 different vector systems, and had been submitted by 100 independent research groups (see Additional file 3). The 575 incompletely trimmed ESTs were grouped according to their cloning vector and the contamination rate relative to the number of sampled ESTs using the same vector for cloning was also calculated (Table 5). Among the 22 vector groups sampled, those ESTs which were cloned into pCMV-SPORT6.1 and pTriplEx2 showed the highest contamination rate (both at around 11%). It is notable that pCMV-SPORT6.1 and pTriplEx2 were almost completely removed from the ESTs by SeqClean (Table 4). Moreover, 174 out of the 186 incompletely trimmed pCMV-SPORT6.1-harbored-ESTs sampled from dbEST (Additional file 3) were derived from the same cDNA library which had been used to study the performance of the three trimming programs in Table 4.

**Table 5:** Vector contamination in ESTs sampled from dbEST

Vector	^a^# Contaminated ESTs	^b^# ESTs	^c^Ratio
pT7T3D-PacI	20	3883	0.52%
pSPORT1	99	3784	2.62%
pCMVSPORT6.0	17	4784	0.36%
pME18S-FL	13	3382	0.38%
pUC18	17	1618	1.05%
pCS107	3	1198	0.25%
PBRcDNASfiIAB	5	1177	0.42%
pBluescriptSK(-)	20	2494	0.80%
pDNR-LIB	24	1817	1.32%
pOTB7	6	891	0.67%
pBluescriptIISK(+)	27	2516	1.07%
pBK-CMV	11	783	1.40%
pBluescriptSK(+)	11	713	1.54%
pExpress-1	19	1507	1.26%
pBluescriptIIKS(+)	7	689	1.02%
pcDNA3.1(+/-)	8	469	1.71%
pCMV-SPORT6.1	186	1676	11.10%
pTriplEx2	60	545	11.01%
pDONR222	20	516	3.88%
pCS108	1	362	0.28%
pT7T3D	1	559	0.18%

Total	575	35363	1.63%^d^

^a^Number of ESTs which were cloned into the indicated vector remained to be contaminated with vector.

^b^Number of ESTs using the indicated vector were subjected to BLAST analysis for identification of vector contamination

^c^percentage of vector contamination for the ESTs using the same cloning vector.

^d^percetage obtained by 575/35363 = 1.63%

In summary, using vectors in CVS form, LUCY2 resulted in the lowest error rate among the three tested programs (Table 3). Though using the vectors in RVS form did not influence the trimming efficiency of LUCY2 or cross_match, RVS forms of the vectors allowed SeqClean to give the best trimming results of all tested programs (see Table 4). Thus we highly recommend our modified procedure to all researchers using SeqClean for the task of vector removal from EST or genomic sequences.

## Discussion

Questions over the quality of the sequence data deposited in the public domain database have caused great concern to life science researchers. Though dbEST provides VecScreen to help check the quality of submitted data, and the Gene indices at TIGR also filter EST data for further grouping, the quality of EST data is still not perfect, even in such a reputable data repository. Previous studies [3,4] reported vector contamination rates of 0.28% to 0.36% for nucleotide sequences in GenBank. In this study, not only was significant vector contamination found in dbEST by analyzing randomly sampled EST data (see Results), but incomplete annotation of ESTs was also observed in both dbEST and Trace Archive. The former error, that is, presence of vector sequences in the ESTs, may cause assembly artifacts as well as errors in many other analyses depending on ESTs, while the latter error affects the amount of effective data for further mining.

This study re-examined the vector contamination rate in the data randomly sampled from dbEST. By looking at the 35363 ESTs (drawn at every 601^st ^entry) which had been cloned into the 22 most prevalent vectors in the dbEST, 575 (1.63%) ESTs were still contaminated with vector sequences. One concern here was that the sampling procedure used here may result in an overrepresentation of the data submitted by very large sequencing projects. If data in such giant projects were incompletely trimmed, errors that existed in the data from other small projects may be overwhelmed and become invisible in the sampled data. In that case, an alternative sampling strategy should be considered. In our test dataset, the 35363 sampled ESTs used were submitted by more than 800 research groups, while the 575 contaminated ESTs were submitted by 100 of them. Furthermore, 174 (30%) of the 575 contaminated ESTs were "contributed" by one single group.

Our study here supports previous research [28] that contamination found in the GenBank data mainly existed in batches submitted by specific groups rather than being stochastically distributed (Additional file 3), suggesting the errors were derived from certain method(s). Yet, it can also be argued that the contamination may be more submitter-dependant than method-dependant. Poor data quality could be caused by various reasons including wet-laboratory practice and bioinformatics processing. However, no matter what the cause, any good vector trimming procedure ought to be able to remove as much contamination as possible.

An effective vector trimming procedure in addition to an efficient confirmation process are thus necessary prior to and during data submission to the public database, and are crucial factors in the reliability of further investigations. Though numerous well-known bioinformatics tools have been made freely available for specific data processing or analysis tasks, each has specific strengths and weaknesses.

An initial vector trimming task revealed a design flaw with the SeqClean program. The trimming errors with this program would occur if the size of the cDNA insert was so small that the start of the vector fell into the critical 30% range from either end of the EST sequences (Figure 1). Hence a vector like pTripleEx2, for example, whose MCS was located far from the start point (Table 2), would be unlikely to be affected by the design flaw of SeqClean. ESTs derived from a cDNA library of mostly long-insert clones may avoid encountering such errors altogether. This flaw was later almost completely overcome with the use of the modified vector form.

When errors are found with one program, an intuitively easy solution is to switch to a different program with similar features. However, there is always the possibility that any new program could give errors of a different type. The results presented in Tables 3 and 4 provide good examples.

The problem with SeqClean did not appear to exist in LUCY2 (Table 3), possibly due to the design of a user-provided splice file required by the program which specified the 100 to 150 bp vector sequences upstream or downstream of the ligation site. With the information in the splice file, the program can accurately anchor the vector sequence around the vector-insert border onto the ESTs in test. However, preparation of an accurate splice file is heavily reliant on users' thorough understanding of the cloning history and careful drafting of the border sequences.

Though cross_match gave a relatively higher error rate than the other two programs tested (Table 3), the length of the vector-matching segments remaining in the processed ESTs were all below 25 bases (Additional file 1). Furthermore, the trimming errors were distributed over various vectors. This implies that the incompletely trimmed sequences were more likely to result from the sensitivity of the algorithm than from an implementation error. Hence, it is probable that the trimming accuracy of cross_match could be improved by properly adjusting the parameter settings. As mentioned earlier, cross_match works in a relatively primitive manner despite its ease of use compared with the other two programs. cross_match identifies the vector-similar sequences in the EST without removing them, hence it requires further computer expertise to obtain a final result of trimmed sequences. Certain EST processing packages like PartiGene which incorporates cross_match for vector trimming in their multi-step pipeline would provide downstream processing to remove the unwanted vector-similar segments and to abandon the ESTs trimmed below a set length limit. Such a pipeline may be a good choice for users to accomplish multiple tasks of a specific purpose with one single procedure; however, it would be difficult to control the errors produced by individual programs along the pipeline. In a test with PartiGene using the same nine sets of EST data as the other programs, almost ten times as many ESTs (2014) were trimmed into <100 bases long segments (data not shown) and were finally removed, in comparison with the results shown in Table 3. Further investigation will be required to elucidate whether the atypical result generated by the program Partigene was due to an inherent design requirement of the processing pipeline, or whether it was caused by an implementation error in the post-processing scripts for cross_match alignments.

To correct an error found in a computer program, users with computer expertise may be able to modify the program code if the source code was made available. Nonetheless, fixing a computer program for re-implementation is often a tedious, uninteresting and error-prone job requiring extensive rewriting and debugging of someone else's code. Therefore we decided to take an alternative strategy by reforming *in silico *the linear form of the cloning vector with a simple word processing program like Notepad or Word. This allows the vector contamination to then be removed from the ESTs with SeqClean (against the reformed vector sequence). The resulting product is evaluated with BLAST.

Though only vector sequences are used for BLAST analysis, short appendix sequences like linkers, adaptors or primers should not affect the result of this confirmation process. As long as the boundaries of the vector sequence are correctly identified, the short sequences will be removed with the vector. On the other hand, if vector does get overlooked by the trimming program, the short sequences will remain untrimmed. This modified procedure for vector trimming clearly corrected almost all of the errors originally detected (Tables 1 and 3).

This procedure, which requires almost no program implementation, may be applicable to a lot of wet-lab based life-science research teams, who are interested in making use of the vast amount of EST data in the public domain but may lack extensive computer expertise. To simplify re-linearization of the cloning vector, a web-based DNA linearization program to pre-process the vector sequences was implemented, which is now available for free on the internet [29]. BLAST evaluation of the performance of the vector trimming (Tables 2 and 3) confirmed that the vector re-linearization step turned SeqClean into an ideal tool for removal of vector contamination from automated sequencing data.

Therefore, we believe that the data quality in the public sequence databanks could be greatly improved if our modified procedure for vector removal was applied to data generated by the majority of high-throughput DNA sequencing projects prior to data submission.

## Conclusion

Vector contamination remains a serious concern to the data quality in the public sequence database. This study identified that a modified procedure using RVS vector form could almost completely correct a design flaw found in SeqClean. The modified procedure, however, had little effect on the other two programs tested, LUCY2 and cross_match. When using vectors in CVS form, LUCY2 performed better than the other two programs under the same test conditions. SeqClean with RVS surpassed the other two trimming programs among all the tested conditions (CVS or RVS), and as it is so easy to use, SeqClean is highly recommended to all researchers for the task of vector removal from EST or genomic sequences.

## Methods

### Data acquisition

EST sequences used in this work were acquired from three different resources. The EST sequences, with or without trace data available, used to study the effect of SeqClean in the initial and the final tests were derived from tomato cDNA clones prepared for research on the stress biology of plants by research laboratories at Agricultural Biotechnology Research Center, Academia Sinica (ABRC). Detailed molecular information about these sequences is stored at TSED (tomato stress EST database) [26]. To compare the three computer programs for vector trimming, in addition to the ABRC ESTs, trace data in SCF format for ESTs were retrieved from Trace Archive at NCBI (June-12-2007 freeze) through the web interface. A total of 530,899 EST entries in Trace Archive [25] were found to contain all the required information for phred processing, including CHEMISTRY, CHEMISTRY_TYPE and CVECTOR. Finally, three cDNA libraries (ZM_BFC, GISZF001_ra and ISUM4) were selected for download. 3000 entries of the EST trace data were randomly drawn from each of the three cDNA libraries using a random number generator. To study the vector contamination rate with dbEST, the entire database of dbEST (2007/4/18 freeze), consisting of 42.3 million entries stored as 604 files, was firstly downloaded from the ftp server at NCBI and then parsed for the vector information recorded in each of the entries. ESTs were sampled at every 601^st ^entry from the files of dbEST and a total of 48212 entries were collected. If an entry without vector information was encountered at sampling, it was removed immediately from the sampled collection (see below).

Around 14.4 million of the dbEST entries were found to contain no vector information and 118 of the 604 files were found to completely lack vector data. The vector information parsed out of dbEST was first grouped manually by synonyms. The number of ESTs under each vector was then counted. Around 74% of ESTs in dbEST were cloned into 22 vectors (Table 5). To investigate vector contamination by specific vectors, the EST entries with vector information among the 22 vectors were drawn from the 48212 sampled ESTs.

### Vector trimming with SeqClean, LUCY2 or Cross_match and result evaluation

Those sequences whose trace data were available were firstly cleaned with the program phred prior to vector trimming by one of the three programs SeqClean, LUCY2 or cross_match. For phred cleaning, a -trim_alt option was included in the command line. Following vector trimming by the indicated bioinformatics program, the processed ESTs were subjected to BLAST analysis against the sequence of their cloning vector, as in Table 2. The highest scoring result for each BLAST analysis was parsed out for further filtering, with criteria based on the method described by Seluja and co-workers [3]: (1) vector sequence occurs at either end of the EST, (2) the matched stretch is longer than 12 bases, and (3) shares cloning vector sequence identity of >95% for those EST pre-cleaned with Phred.

To examine the error rate in dbEST sequences from NCBI, the same criteria described above were applied to parse the BLAST output. Error rate of EST in this study indicates the ratio of ESTs which contained residual vector sequences (>12 base pairs) even after processing by a vector trimming program to the number of all the EST sequences subjected to the same trimming process.

Parameters were set at default values for SeqClean and LUCY2. For cross_match processing, a more sensitive setting (-minmatch 10 and -minscore 20) than default was applied, as recommended in the user manual.

For the LUCY2 trimming, in addition to files of EST or vector sequences, splice files were prepared as input data. A splice file contains two pairs of sequences: up to 150 base long sequences upstream or downstream of the cloning site on the vector in either orientation. Two splice files were prepared for each cloning vector, one for CVS and the other for RVS. The sequences in the splice files made from CVS or RVS of one cloning vector were basically identical except that the extended lengths derived from pDNR-LIB, pGEM-Teasy and pT7Blue were a little shorter for CVS than RVS since their splice sites fall within 100 bases from the start points of the vector sequences. The splice sequences for the three sets of ESTs derived from Trace Archive (i.e. those which were cloned into pCMV-SPORT6.1, pT7T3-PAC, and pDNR-LIB) were produced based on the library description documented in the dbEST. For the rest of the tested ESTs which were produced in our facility, the splice sequences were generated according to the commercial or the laboratory protocols for the cDNA library construction procedure and were later confirmed with the technician who performed the experiments. Prior to LUCY trimming, Bl2seq (a BLAST variant for pairwise sequence alignment) was used to double-check the generated splice sequences for the accuracy of the sequences and orientations.

The bioinformatics programs used in this study, including cross_match (version 0.990329), PartiGene (version 3.0.3), phred (version 0.020425.c), LUCY2 (version 2.19p-R07), SeqClean (downloaded on 07/13/2004), and BLASTN (version 2.2.15) were all obtained through their official websites. Large-scale data processing with phred, SeqClean, cross_match, PartiGene and BLAST programs was carried out at command line level on a Linux platform, while analysis with LUCY2 was performed on a Windows XP platform. Integration of the processed data and further analyses were performed with the programs Excel and Access on a Windows XP platform.

The web-based DNA linearization program was implemented in php language. Parsing of the trimming information was performed using a program written in perl. Batch BLAST was carried out with command lines on a Linux platform using the BLAST program downloaded from NCBI. The BLAST results were also parsed with an in-house perl program.

## Authors' contributions

YC participated in data analysis, hypothesis formation and helped to draft the manuscript. CL carried out most of the program implementation and analysis for vector trimming and BLAST evaluation on the trimming results. CW performed the initial SeqClean trimming. HW conducted PartiGene analysis and studied usage of LUCY2 and cross_match prior to data analysis. PH conceived of the study, participated in its design and coordination and drafted the manuscript. All authors read and approved the final manuscript.

## Supplementary Material

Additional file 1 BLAST evaluation on the vector trimming results conducted with the three trimming programs using either vector form. The BLAST analysis results following the filtering criteria used through this report are shown in this Excel file. The bioinformatic program and the vector form used for trimming are indicated in the name of the worksheet. The worksheet "column descript" provides a description of what each column name represents. This file contains all the source data used to derive Tables 3 and 4 in the main text. Click here for file

Additional file 2 Effect of vectors on vector trimming performance by three programs. The same as Table 4 in the main text except that the number, instead of the percentage, of incompletely trimmed ESTs was used. Click here for file

Additional file 3 Artifact vector trimming found with ESTs from dbEST at NCBI. Error rate of dbEST with emphasis on vector contamination was investigated by "BLASTing" the ESTs randomly sampled from dbEST at NCBI either against the UniVec (worksheet "601_UniVec") or against the sequences of their cloning vectors (worksheet "601_22vector"). Shown in the Excel file are the filtered BLAST results according to the criteria described in Methods. Please note that in worksheet "601_22vector", only 35,363 EST sequences which were cloned into the most prevalent 22 vectors were used for BLAST analysis (Please see methods for details.) The Spreadsheet "col des" provides a description of each column. Click here for file
