# Supplementary material for: An optimized procedure greatly improves EST vector contamination removal
Source: BMC Genomics. 2007 Nov 13;8:416. doi: 10.1186/1471-2164-8-416 (PMC2194723; doi:10.1186/1471-2164-8-416)
Supplement: Additional file 2 — Effect of vectors on vector trimming performance by three programs. The same as Table 4 in the main text except that the number, instead of the percentage, of incompletely trimmed ESTs was used. [file 1471-2164-8-416-S2.pdf]

Supplement Table 1- Effect of vectors on vector trimming performance by three programs

Vector	#EST tested	Trimming Programs							
		SeqClean		**CrossMatch**		LUCY2		LUCY2-qul	
		CVS	RVS	CVS	RVS	CVS	RVS	CVS	RVS
PCMV.SPORT-6.1	3000	1	1	95	95	9	8	5	5
PT7T3PAC	3000	0	0	7	23	3	2	1	1
PDNR-LIB	3000	9	9	35	35	0	0	1	1
pGEM-Teasy	2882	1106	1	186	206	15	15	9	9
pTriplEx2	2318	1	1	224	224	108	113	123	128
pCR21TOPO	5	0	0	0	0	0	0	0	0
pZL1	700	10	0	27	27	19	19	16	16
pT7Blue	130	1	0	12	12	1	33	1	33
Total		1128	12	586	622	155	190	156	193
